# Plant Material in the Thalamus and Mesencephalon of a Young Dog

**DOI:** 10.3390/vetsci12020076

**Published:** 2025-01-21

**Authors:** Maria Winnerby, Tove Nielsen, Ellen Andersson, Cecilia Rohdin

**Affiliations:** 1 Anicura Djursjukhuset Albano, Rinkebyvägen 21B, 18236 Danderyd, Sweden; 2 SVA—Statens Veterinärmedicinska Anstalt, Ulls väg 2B, 75189 Uppsala, Sweden

**Keywords:** foreign body, thalamus, mesencephalon, plant material

## Abstract

An unusual case of neurological deficits due to intracranial plant material in the thalamus is presented. The case was a young dog with pyrexia, neck pain, and neurological deficits that were consistent with an intracranial multifocal lesion. Based on the imaging findings and the result of the cerebrospinal fluid analysis, a presumptive diagnosis of immune-mediated meningoencephalitis was made. The dog initially responded to immunosuppressive treatment but then deteriorated and was euthanized. At autopsy, plant material was found in the thalamus and mesencephalon. Intracranial foreign bodies are unusual but should be considered as a differential diagnosis in young dogs with neurological deficits that are consistent with a focal or multifocal intracranial lesion.

## Simple Summary

Intracranial foreign bodies are rare in veterinary medicine, with only fourteen published cases having been reported in dogs. Of these, half involved intracranial plant material. This case report describes a young dog with plant material being found in the thalamus and mesencephalon. Initially, the dog was misdiagnosed with immune-mediated meningoencephalitis and treated with immunosuppressive doses of prednisolone. Although the dog initially responded to the treatment, its condition later deteriorated, leading to euthanasia. An autopsy revealed the presence of intracranial plant material. This case highlights that while intracranial plant material is uncommon, it should be considered an important differential diagnosis in young dogs presenting with neurological deficits that are indicative of focal or multifocal intracranial lesions.

## 1. Introduction

Few cases of intracranial foreign bodies have been reported to date. In the fourteen cases reported, needles [1,2,3,4], a wooden cocktail stick [5], porcupine quills [6,7], and plant material [8,9,10,11,12,13] have been identified (Table 1). Although the route of entry is often obscure, foreign bodies may enter the cranial cavity through perforations of the oral cavity, the nasal cavity, the ear canal, and the orbits [14]. 

The clinical signs associated with intracranial foreign bodies are caused by their mere presence or by introducing bacterial agents that cause an abscess [4,9] or diffuse bacterial encephalitis [8]. The clinical signs can therefore have an acute (diffuse encephalitis) or a more chronic (abscess) presentation (Table 1). Plant materials, especially grass awns, are a major contributor to foreign body disease in dogs, and the migratory capacity of these foreign bodies can precipitate a variety of syndromes [8,14]. The antemortem diagnosis of migrating foreign bodies, specifically small objects without an apparent migratory tract, can be challenging, as the foreign body may not be visible in studies obtained with diagnostic imaging techniques [9]. It has therefore been suggested that cases of canine regional encephalitis in which a primary cause or septic focus cannot be identified should be closely examined for foreign plant material [8].

## 2. Case Description

A 5-month-old, female, 5 kg, mixed breed was presented to Anicura Albano Small Animal Hospital for evaluation of depressed mental status, pyrexia (40.5 °C), and neck pain. The clinical signs began one day prior to presentation (day 2). The physical examination confirmed the dog’s depressed mentation and neck pain but not pyrexia (38.2 °C). In the neurological examination, the dog showed depressed mentation, head turn to the left, compulsive walking, and circling to the left. The postural reactions were decreased in the right thoracic and pelvic limbs, with intact spinal reflexes. The menace response was depressed bilaterally, and there was bilateral positional ventral strabismus. Based on the results from the neurological examination, the neurolocalization was consistent with an intracranial multifocal lesion involving the thalamus and/or left forebrain. 

**Figure 1:**
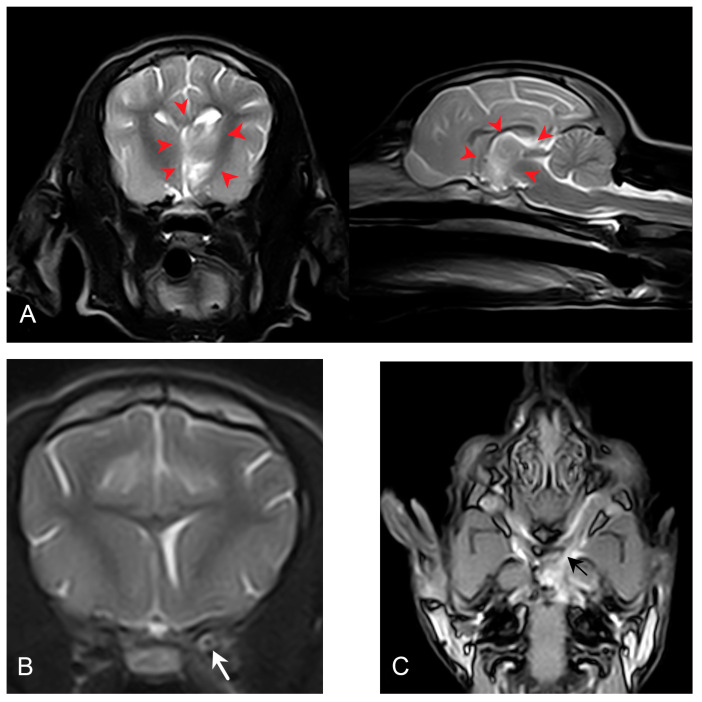
Sagittal (**right**) and transverse (**left**) T2-weighted MRI images demonstrating part of the diffusely outlined intraparenchymal T2W hyperintensity within the left thalamus, hypothalamus, and rostral part of the mesencephalon (red arrowheads). The abnormality is heterogeneously hyperintense in T2W, with streaks of increased T2 signal. There is a mild midline shift but no signs of transtentorial herniation (**A**). Transverse T2-weighted MRI image demonstrating the hyperintensity of the left optic nerve (white arrow) (**B**). Dorsal postcontrast 3DSST1 MRI image demonstrating contrast enhancement of the left optic nerve (black arrow). The left optic nerve is wider than normal, and the rostral limit of the changes is rostral to the left optic canal and extending into the optic chiasm, confluent with severely enhancing meninges around the left piriform lobe, as well as the ventral part of the left temporal lobe (**C**).

The haematology (IDEXX ProCyte Dx, IDEXX Nordics, Solna, Sweden), complete blood count, and biochemistry (IDEXX Catalyst One, IDEXX Nordics, Solna, Sweden) at presentation (day 2) were unremarkable, except for a mildly increased C-reactive protein (CRP) (39.4 mg/L, range 0.0–10.0 mg/L). Magnetic resonance imaging (MRI) and cerebrospinal fluid (CSF) analysis were performed two days after presentation (day 4). During this time, the dog was treated with prednisolone (prednisolonacetate, CP Pharma, Burgdorf, Germany) at 2 mg/kg subcutaneously once daily and received intravenous fluid therapy (Ringeacetate, Fresenius Kabi, Uppsala, Sweden) at 3 mL/kg/h). Treatment was initiated due to a suspicion of immune-mediated meningoencephalitis, based on the clinical presentation suggesting a multifocal localisation. Over the next two days, the dog showed clinical and neurological improvement (improved mentation and disappearance of the head turn), and CRP was decreased to 16.5 mg/L (reference range: 0.0–10.0 mg/L). Magnetic resonance imaging (MRI) of the brain was performed using a standard protocol (T2-weighted, T1-weighted, fluid-attenuated inversion recovery (FLAIR), and T1W3D pre–postcontrast) with a 0.2 Tesla permanent magnet (Esaote Vet-MR Grande, Esaote, Genoa, Italy). The MRI revealed a left-sided intraparenchymal, diffusely outlined abnormality involving the thalamus, part of the hypothalamus, and the rostral part of the mesencephalon (Figure 1). The abnormality was heterogeneously hyperintense in T2W, with streaks of increased T2W signal and focal areas of reduced T1W signal, which were not contrast-enhancing. Besides the intraparenchymal abnormalities, the meninges surrounding the piriform lobe and the ventral part of the left temporal lobe showed contrast enhancement. The left optic nerve was wider than normal, was hyperintense in T2W, and was contrast-enhancing (Figure 1). 

The cerebrospinal fluid (CSF) that was obtained from the cerebellomedullary cistern was cloudy, with a total nucleated cell count of 5200 cells/µL and a marked mixed pleocytosis (1660 cells/µL mononuclear leukocytes; range: 0–5 cells/µL and 3540 cells/µL polynuclear leukocytes; range: 0–3 cells/µL) and increased protein concentration (1.2 g/L; range: 0.00–0.25 g/L). Although warranted by the cell count results, cytology and bacterial cultures of CSF were not conducted. 

A suspected diagnosis of immune-mediated meningoencephalitis was made based on the multifocal abnormalities observed on MRI, and the treatment with prednisolone was continued. The dog was discharged the day after (day 5) diagnostic workup. The dog continued to improve clinically and neurologically, but two days (day 7) after discharge from the animal hospital, the dog deteriorated, showing depressed mentation, compulsive walking, head turn to the right this time, and seizures. At the owner’s request, the dog was humanely euthanized and sent for autopsy. 

**Figure 2:**
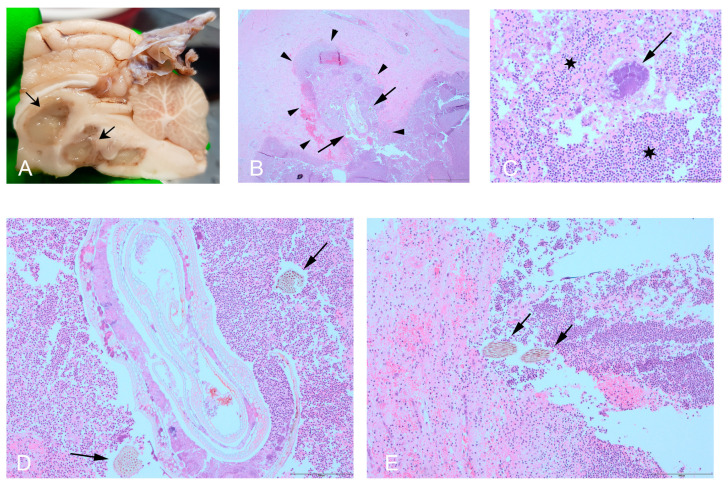
Gross pathology and histopathology of the brain. Gross examination demonstrating multifocal-to-coalescing purulent areas (arrows) in the white matter of the central and caudal parts of the brain (**A**). Histopathology of the thalamus, adjacent to the hippocampus, demonstrating plant material (arrows) in the centre of a pyogranuloma (arrowheads). The pyogranuloma was primarily composed of neutrophils but also macrophages, H&E20× (**B**), and H&E100× (**D**). Numerous bacterial colonies (arrows) at the centre of a pyogranuloma in the left thalamus. Note the surrounding severe inflammation consisting of neutrophils and macrophages (stars), H&E200× (**C**). Foreign material of a plant origin (arrows) surrounded by pyogranulomatous inflammation and haemorrhages in the left mesencephalon, H&E100× (**E**).

Macroscopically, a midline sagittal incision into the central parts of the brain revealed multifocal-to-coalescing purulent severe leukomalacia (Figure 2). The greenish-grey purulent lesions in the white matter were mainly observed in the left side of thalamus but were also present in the right side of the thalamus, as well as the hippocampal region, mesencephalon, cerebellum, and adjacent parts of the medulla oblongata. The histopathology of the thalamus, mesencephalon, cerebellum, and medulla oblongata showed multifocal pyogranulomas composed of leukocytes, primarily neutrophils and macrophages (Figure 2). At the centre of the pyogranulomas, there were bacterial colonies, and in the thalamus and mesencephalon, variably sized (64–1127 µm) and shaped areas of foreign bodies, consistent with plant material, were noted to be associated with these colonies. 

## 3. Discussion

Intracranial foreign bodies are rare but important differential diagnoses to consider in dogs presenting with pyrexia and neurological signs that are consistent with intracranial lesions. Although the clinical picture, together with the results of the MRI and specifically the CSF, should have raised a suspicion of bacterial encephalitis, confirming the presence of intracranial foreign bodies antemortem would have been challenging in this case. 

Plant material, particularly grass awns, is the most common finding in dogs with intracranial foreign bodies and is reported in half of these cases (Table 1) [8,9,10,11,12,13]. Plant material is thought to enter the brain either through penetration of the cribriform plate (nasal migration), through perforation of the pharyngeal soft tissue and basisphenoid bone (oropharyngeal migration), by penetration through the foramen magnum, through orbita (periorbital migration), or by severe penetrating trauma through the skull bone. However, the migration routes of foreign bodies in the CNS often remain unknown [8,9]. In the majority of previously presented cases, the route of entry was unknown (Table 1). In two dogs, there was a suspicion of entry of the plant material through periorbital migration [10,11]. Due to the contrast enhancement of the left optical nerve on the MRI, entry by this route appears likely in this case as well, although no signs of inflammatory changes to the optic nerve were seen on the histopathology. The absence of inflammatory changes in and around the optic nerve on the histopathology could be due to the immunosuppressive treatment during the four days between the MRI examination and autopsy. Intracranial penetration is not a rare complication of periorbital wooden foreign bodies in humans, especially in smaller children [15]. The neurological manifestations do not always present immediately, and as a result, the foreign body may go undetected for some time, allowing it to migrate [16]. A delay of neurological symptoms has also been seen in dogs with intracranial plant material [8,9,11], and maybe that is the reason for an absent visible route of entry and migration. Foreign bodies like plant material and grass awns easily collect in the conjunctival sac of dogs running outside, and mild conjunctivitis caused by a foreign body may easily go undetected by owners, which makes the periorbital route a possible route of entry for plant material in dogs [14]. 

In the majority of dogs who presented with intracranial plant material, the diagnosis was established post-mortem (Table 1), and only two have successfully been treated surgically [10,12]. The intracranial plant material was visible in only one previously reported case [12], where an MRI was performed. The visibility of a foreign body on MRI depends on its size and hydration, the composition of surrounding tissue, and the inflammatory response. Even if the foreign body is not directly visible in MRI, the surrounding inflammation should be reliably detectable [17]. In two previously reported cases of dogs with intracranial plant material [10,12], a CT scan of the skull was performed. In one case, the plant material appeared as a linear hyperattenuating structure, while in the other case, it was not visible. Plant material can present as either hyper- or hypointense on CT, and if sufficiently large, it may be detected. However, plant material that is surrounded by body fluid or with inherent soft tissue attenuation may not be detectable on CT [18]. In both cases in which intracranial plant material was present, and a CT scan was performed, an osseous defect in the calvaria was identified, suggesting a potential route of entry [10,12]. In our case, the plant material was not visible on the MRI, not even when re-evaluated after receiving the autopsy report. Probably, the small size of the plant material, together with the thickness of the slices and the fact that a low-field magnet was used for the MRI examination, contributed to why the plant material was not visible. In our case, a CT scan of the skull could have provided valuable information, such as identifying an osseous defect and a potential route of entry. Ultrasound is a useful tool for diagnosing grass awns and plant material, as these foreign bodies typically appear hyperechoic with distal acoustic shadowing artefacts [19]. For intracranial plant material to be visualized by ultrasound, there needs to be an open fontanelle. However, the plant material in our case was likely too deeply embedded in thalamus to be visualized by ultrasound, even during surgical exploration.

In our case, as well as in three previously reported cases of intracranial plant material, the dogs were treated with prednisolone for suspected immune-mediated meningoencephalitis or steroid-responsive meningoarteritis (SRMA) [8,9,13]. Initially, our dog and two others responded to prednisolone treatment but later deteriorated and were euthanized. The initial response was likely due to suppression of the inflammation. Following the initial positive anti-inflammatory response, the immunosuppressive treatment likely caused an aggravation of the infection that was introduced with the plant material, leading to a deterioration of neurological signs. 

Intracranial bacterial diseases are rare in veterinary medicine, with the spread of bacteria from otitis media being the most common cause [20,21]. Immune-mediated causes of meningoencephalitis are considerably more common than infectious conditions [20], and they are usually widely spread throughout the CNS [22]. The majority of dogs with immune-mediated meningoencephalitis are small-breed dogs, and although they are commonly presented as adults, young dogs are reported in the literature [23]. This knowledge, together with the MRI findings of a multifocal intra-axial lesion involving more than one region of the brain and the fact that there was no history of trauma, including bite wounds, or signs of otitis media/interna, were contributing factors underlying the decision to treat our dog for an immune-mediated meningoencephalitis. Although pyrexia can be seen in both immune-mediated meningoencephalitis and infectious causes of encephalitis [20], pyrexia at a very young age, as in this case, and the severe pleocytosis should have raised a suspicion of an infectious cause. The treatment with prednisolone in our case was questionable and based on the CSF, cytology and bacterial cultures should have been performed. 

To our knowledge, there are only two dogs with intracranial plant material that have been successfully treated with surgery [10,12]. In the remaining cases, the progression of the disease has been too acute, or, as in our case, treatment has been inadvertently delayed due to misdiagnosis, making surgery no longer an option. Two previously reported cases of dogs with intracranial plant material were treated with antibiotics due to a suspected infection, but both these cases deteriorated and were euthanised before diagnosis [8,11]. In our case, the multiple small widespread pieces of plant material, located deep in the brain, would have been very challenging to localize and surgically remove, even with a timely diagnosis. Treating the dog with antibiotics might have temporarily suppressed the infection, but it likely would have recurred once the antibiotics were discontinued. 

## 4. Conclusions

Intracranial foreign bodies are rare but should be a differential diagnosis when young dogs present with neurological signs of a focal or multifocal intracranial lesion. A definitive diagnosis of an intracranial foreign body is challenging in the absence of clinical suspicion.

## Figures and Tables

**Table 1:** Signalment and variables of previously published cases of dogs with intracranial foreign bodies.

Breed	Age	Type of Foreign Body	Intracranial Location	Route of Entry	Clinical Signs	Diagnostics	Outcome
Cavalier King Charles Spaniel [1]	11 month	Sewing needle	Right neurocranium	Oral, through the foramen lacerum	Pyrexia. Hypersalivation, chewing. No neurological signs.	Skull radiographs	The foreign body was surgically removed, and the outcome was good
Mixed breed [4]	12 year	Sewing needle	Left piriform lobe and lateral thalamic nuclei	Oropharyngeal migration	Five-day history of inappetence, falling over to the right, ataxia, tetraparesis, and decreased mental status.	Pathology	Died
Maltese [2]	8 year	Sewing needle	Right temporal cortex	Oropharyngeal migration	Cluster seizures after a minor car accident 3 days before presentation.	CT	The foreign body was surgically removed, and the outcome was good
Maltese [3]	1 year	Sewing needle	Brainstem, cerebellum, and caudal part of forebrain	Nasopharynx	Acute hemorragical vomiting and seizures.	CT	The foreign body was surgically removed, and the outcome was good
Terrier mixed breed [8]	5 month	Grass awn	Right lateral and third ventricles	A definitive foreign body migratory tract was not grossly evident	Pyrexia, acute-onset tetraparesis, depressed mentation.	Pathology	Died
Great Dane [8]	4 month	Grass awn	Right occipital lobe, internal capsule, lateral ventricle	A definitive foreign body migratory tract was not grossly evident	Acute-onset tetraparesis, depressed mentation, tonic–clonic muscular contractions.	Pathology	Died
Brittany Spaniel [8]	4 year	Plant material	Right occipital lobe, lateral ventricle	A definitive foreign body migratory tract was not grossly evident	Acute-onset ataxia, depressed mentation, and head pressing.	Pathology	Initially responded to steroid treatment, deteriorated, and was euthanized
Dachshund [9]	7 month	Plant material	Medulla oblongata	A definitive foreign body migratory tract was not grossly evident	One-month history, ataxia, tetraparesis, and depressed mentation.	MRI brain	Euthanized
Great Dane [10]	3 year	Grass awn	Left internal capsule at the level of the optic canal	Through the bone dorsal to the orbital fissure	Two-week-onset behavioural changes, 24 h onset seizures.	MRI brain and CT	The foreign body was surgically removed, and the outcome was good
Hungarian Vizla [11]	22 month	Plant material	Caudal portion of the right cerebral hemisphere	Probably a periocular migration route	Five-day history of pyrexia of an unknown aetiology. The dog was treated with antibiotics, but 20 days later, it developed seizures.	MRI brain	Died
Hungarian Vizla [12]	4 month	Grass awn	Right cerebral hemisphere	Through the rostral right preshenoid/basosphenoid junction	Two-day history of lethargy, inappetence, vomiting, neck pain, and seizures.	CT and MRI brain	The foreign body was surgically removed, and the outcome was good
West Highland White Terrier [13]	2 year	Plant material	Left caudal colliculus and rostral cerebellar hemisphere	Unknown	Acute neck pain initially and three months later seizures.	MRI spine	Initially responded to steroid treatment, deteriorated, and was euthanised
Golden retriever [6]	2 year	Porcupine quill	Left cerebral hemisphere	Soft palate, nasopharynx, and left oval foramen to the neurocranium	Halitosis, lethargy, ptyalism, recurring oral haemorrhages, gagging, and episodes of seizures.	CT	Initial response to antibiotic treatment, deteriorated and was euthanized
St Bernard [7]	Adult	Porcupine quill	Left occipital lobe	Probably through foramen magnum	Progressive severe neurological signs. Three months earlier, the dog had attacked a porcupine.	Pathology	Euthanasia
Border collie [5]	4 year	Wooden cocktail stick	Right parietal, frontal, and temporal lobes	Oropharyngeal migration	Ten-day history. Intermittent lethargy, inappetence, retching, dysphonia; later, depressed mentation and behaviour and an episode of collapse.	CT and MRI brain	The foreign body was surgically removed, and the outcome was good

## Data Availability

This article includes all relevant study data.
